# A minireview of cholera outbreak in Lebanon – a rising public health concern

**DOI:** 10.1097/MS9.0000000000000293

**Published:** 2023-03-25

**Authors:** Christin Berjaoui, Nourhane Al Akoum, Ahmad El Nouiri, Saadeddine Khayat, Mortada Abbass, AlHareth Al Mousawi, Jack Wellington, Olivier Uwishema

**Affiliations:** a Oli Health Magazine Organization, Research and Education, Kigali, Rwanda; b Faculty of Medicine, Beirut Arab University, Beirut, Lebanon; c Cardiff University School of Medicine, Cardiff University, Cardiff, UK; d Clinton Global Initiative University, New York, New York, USA; e Faculty of Medicine, Karadeniz Technical University, Trabzon, Turkey

**Keywords:** cholera, Lebanon, outbreak

## Abstract

Cholera is a highly contagious illness that can cause severe, acute, watery diarrhea. The WHO and the Lebanese Ministry of Health announced on the 10 October 2022 the re-emergence of Cholera in Lebanon. Data was collected from the Ministry of Public Health in Lebanon, the WHO, news announcements, as well as from online databases such as PubMed, Science Direct, news, conferences, and press releases on the current cholera outbreak. More than 669 confirmed cholera cases and 23 deaths have been reported in Lebanon up until 29 December 2022. The Ministry of Public Health is providing cooperation and support in containing the disease and covering the hospital and treatment expenses for cholera patients. This paper aims to study the epidemiology of cholera, focusing on the most recent cholera outbreak in Lebanon, and to suggest some recommendations that can be followed to fight off this outbreak.

## Introduction

Highlights

- The WHO and the Lebanese Ministry of Health announced on the 10 October 2022 the re-emergence of cholera in Lebanon.
- More than 390 cholera confirmed cases and 17 deaths have been reported in Lebanon.
- The ministry of public health is providing cooperation and support in containing the disease and covering the hospital and treatment expenses for cholera patients.

When *Vibrio cholerae* bacteria are present in food or water, it can result in the acute diarrheal illness known as cholera. Public health continues to be affected by cholera, which also serves as a sign of inequality and a lack of social advancement^1^.

Cholera can be acquired via consuming infected food or drinking water, through the feco-oral route. When there is an epidemic, the water or food is typically contaminated by an infected person’s excrement. In locations where sewage and drinking water are not properly treated, the illness can spread quickly. Casual contact with an infected individual is not a risk factor for getting sick because the bacteria are unlikely to be transmitted directly from one person to another^2^.

Ending Cholera: A Worldwide Roadmap to 2030, a global Cholera control strategy, was introduced in 2017. Its goal is to reduce cholera mortality by 90%^3^.

With the current conflict in nearby Syria, cholera is making a comeback in a collapsing Lebanon, where the Gross Domestic Product (GDP) growth rate reached −21.4 in 2020, and −7 in 2021 as reported by the World Bank^4^. The WHO and the Lebanese Ministry of Public Health announced on the 10th of October the re-emergence of cholera in Lebanon, with more than 699 cases reported so far up until 29 December 2022^5^.

This paper aims to study the epidemiology of cholera, focusing on the most recent cholera outbreak in Lebanon, and to suggest some recommendations that can be followed to fight off this outbreak.

## Microbiology and pathophysiology

*V. cholerae* is a highly motile, single-flagellated facultative anaerobe, a gram-negative rod comma-shaped bacteria*. V. cholera* is naturally found in aquatic environments, this bacteria is salt tolerant and requires NaCl for growth. The strains that are associated with cholera are the toxigenic ones^6^. With more than 200 serogroups and based on phenotypic distinctions, only two serogroups, O1 and O139, are associated with cholera, with O1 being classified into two strain biotypes, which are the classical and El Tor^6^,^7^. *V. cholerae* is usually an asymptomatic, mild infection. However, *V. cholerae* may also lead to severe, life-threatening diarrhea if not treated well^6^.

**Figure 1:**
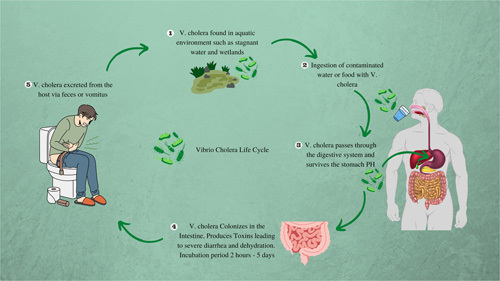
The life cycle of *Vibrio cholerae*. PH, potential of hydrogen.

*V. cholerae* colonizes in the small intestine through the toxin-coregulated pilus after being ingested. Toxin-coregulated pilus also makes the bacteria resistant to bile. The main virulence factor is the cholera toxin^6^ (Fig. 1).

## Epidemiology

Cholera has plagued humans for hundreds, if not thousands, of years, particularly in the areas around the Bay of Bengal, with some of the first records of cholera-like illness reported in ancient Sanskrit medical texts written around 500–400 BCE^8^. Cholera pandemics are the term used to describe the seven times the disease spread past Asia during the 19th and 20th centuries. Seven cholera pandemics have been reported, with the first one beginning in 1817. Although the patterns of worldwide expansion have taken many different shapes, they have generally influenced much of Asia, Africa, Europe, and the Americas at some point or another^9^. Based on phenotypic distinctions, two O1 strain biotypes – classical and El Tor – were identified^7^. The first six of the seven major cholera pandemics that have occurred since the beginning of the 19th century were caused by classical strains; however, the seventh pandemic, which broke out in 1961 on Sulawesi, an Island governed by Indonesia, and extended to Africa in 1971 and the Americas in 1991, was caused by El Tor strains. In 1992, serogroup O139 appeared in Indian subcontinent and Bangladesh expanded throughout Asia, but it was eventually replaced by O1 strains that are still causing cholera till this day^7^.

## Return of cholera to Lebanon

After the novel coronavirus disease 2019 pandemic hit worldwide and exhausted the health system, an economic crisis struck Lebanon, sending it into freefall. In addition to this, the increase in low socioeconomic status made this country an easy target for outbreaks and the re-emergence of many communicable diseases. One of these diseases, cholera, has made a comeback after the last outbreak in 1993^10^.

In 1993, cholera started in the neighboring country of Syria and crossed into Lebanon, causing many cases but few deaths; it lasted for about 3 months^11^.

The Syrian crisis affected the Lebanese health system in many ways as well. Lebanon has been welcoming a large number of refugees ever since the crisis began. Thus, increasing the population by 30% further burdened this resource-limited country^12^. This constant influx of refugees has played a role in the transmission of cholera across the border. The shared river between Lebanon and Syria has also served as a vehicle of transmission across the border given cholera’s water-borne nature.

**Figure 2:**
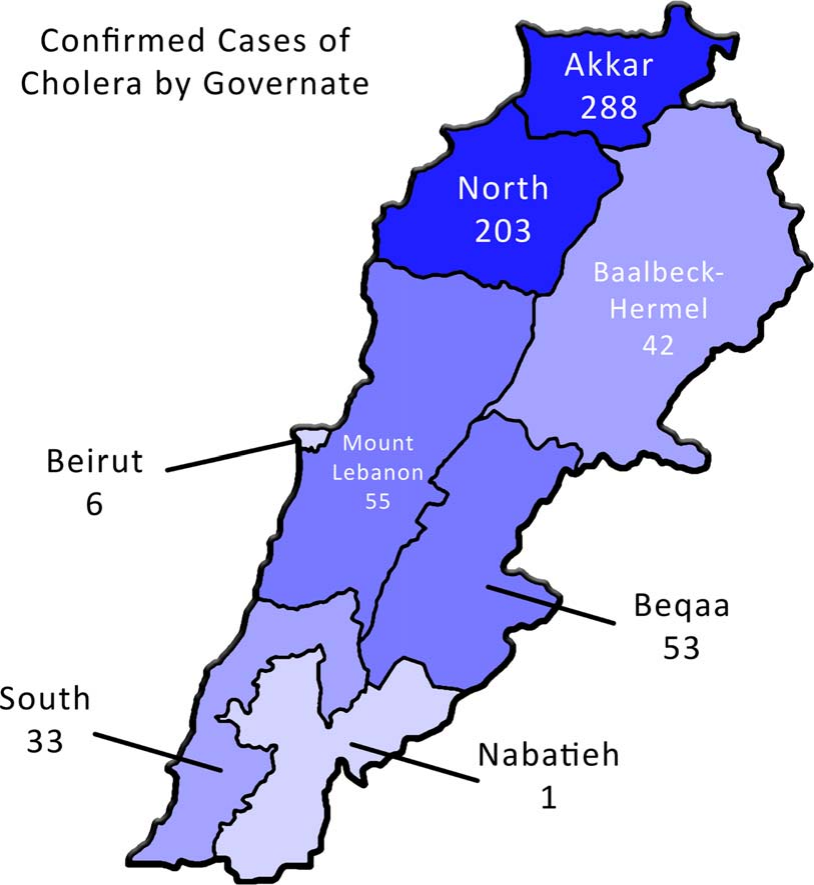
Map of Lebanon showing the distribution of cholera confirmed cases across the country.

As of 29 December 2022, the Lebanese Ministry of Public Health has reported a number of infections, 669 cases, and 23 deaths^5^. The Lebanese people and government are now confronted with the threat of the spread of cholera, considering most refugees use tap water as a source of drinking water^13^,^14^. The outbreaks were reported not only in areas with a higher refugee concentration but also in other Lebanese regions (Fig. 2). This was attributed to a lack of immunizations, a low socioeconomic status, and poor living conditions^12^.

## Preparedness to fight off cholera in Lebanon

The economic crisis Lebanon is facing has imposed many burdens on the healthcare system including lack of equipment and labor shortage. These burdens are manifested by the inability of patients to afford medical services, especially given that 40% of the population is now below the poverty line^15^,^16^.

The Lebanese government has shown support for the victims of this outbreak. On the 12 October 2022 the minister of public health stated that ‘the Ministry of Health will cover all Lebanese patients infected with cholera, international bodies with whom we have reached have expressed their readiness to cover the treatment of displaced people or refugees infected with cholera’^17^.

Moreover, a 20 bed field hospital for patients with cholera started operating in Lebanon^18^. In addition, Lebanon received 13 440 doses of cholera vaccine, which were donated by France^19^. Furthermore, 900 000 cholera vaccines will have arrived in Lebanon by mid-December 2022 with support from the WHO and funded by the United Nations Central Emergency Response Fund and the Contingency Fund for Emergencies^20^.

**Figure 3:**
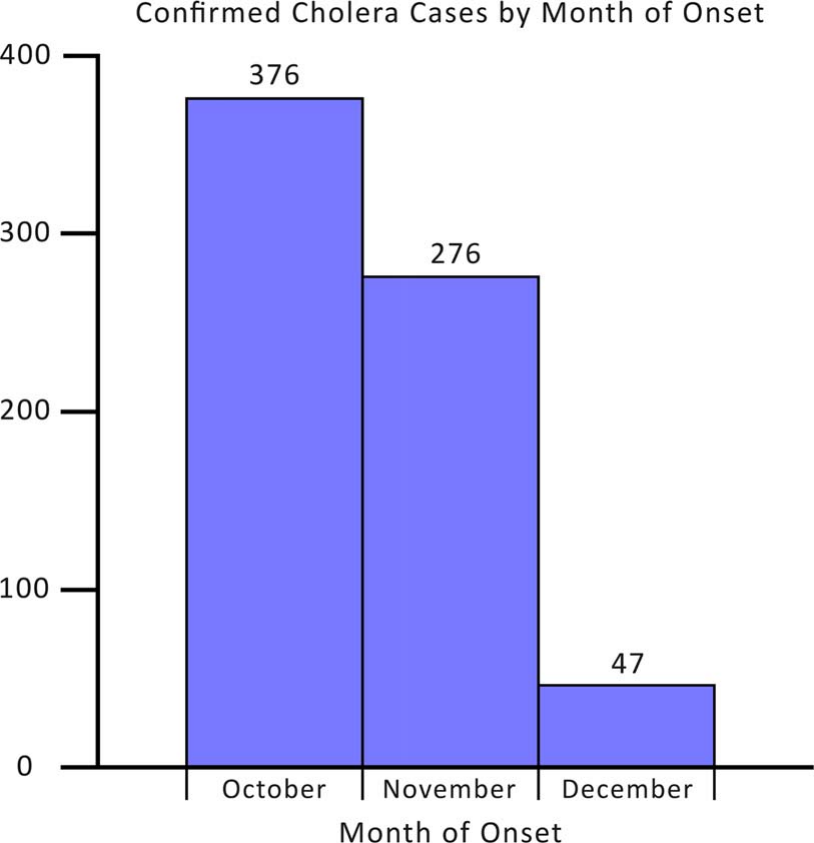
Confirmed cholera cases in Lebanon by month of onset.

With all these measures being taken, a decrease in cholera cases from 376 in October 2022 to 47 in December 2022 has been noted (Fig. 3)^5^.

## Diagnosis and management

As per the WHO the suspicion of cholera is divided accordingly. In communities where an outbreak has been declared, cholera should be suspected when a patient 2 years of age or older presents with acute watery diarrhea and severe dehydration or dies from acute watery diarrhea. When an outbreak gets declared, however, any case or death due to acute watery diarrhea becomes a suspect of cholera infection. The WHO defines a confirmed case of cholera as a positive culture or PCR test for *V. cholerae* O1 or O139 in any suspected case. When cholera is not prevalent in a country, the isolated organism should be proven to be toxigenic^21^.

Rapid diagnostic tests such as Crystal VC, Chokit, and others are also available for cholera diagnosis^22^,^23^.

The management of cholera is focused largely on dehydration. Fluids are administered through oral rehydration solutions. Patients with severe disease and dehydration should be rapidly given fluids through intravenous access^24^. Ringer’s lactate is the recommended fluid; if not available, normal saline can be used^2^.

Antibiotics such as tetracycline, doxycycline, and azithromycin, can be used for severely dehydrated patients and moderately dehydrated patients that exhibit a net fluid loss despite fluid resuscitation^24^,^25^. Many antibiotics have been used and have shown effectiveness^24^,^25^. This has led to the emergence of resistant strains, which is one of the reasons the WHO recommends against the widespread use of antibiotics^24^. In addition to the fact that they have not been shown to decrease spread on a mass level^24^.

The final element of the management is nutrition. Lactating mothers of cholera patients are encouraged to continue breastfeeding concurrently with fluid therapy^2^,^25^. Zinc supplementation helps decrease the duration and severity of the disease. It is administered orally at a dose of 20 mg daily for all children older than 6 months^2^.

In Lebanon, diagnosis and management guidelines are being followed by major hospitals and some governmental hospitals. In the north, where minimal resources are available, a 20 bed field hospital for patients with cholera was established by the Ministry of Public Health^18^. In addition, the donated cholera vaccines that were supplied to the country were distributed to both healthcare workers as well as the general public, particularly refugees that reside in the areas greatly affected by the outbreak^19^,^20^.

## Recommendations

Since cholera prevention is based on sanitary conditions, it is advisable that people wash their hands prior to food handling, drink safe water such as bottled water, water boiled for 1 min or chlorinated water, and eat boiled, freshly cooked, hot food. Avoidance of tap water, ice cubes, raw, undercooked meat, fruits, vegetables, and seafood is also a key. The use of 60% alcohol-based sanitizer may be used as an alternative for water and soap hand washing. On top of that, people, and contacts of family members infected with cholera, are advised to seek medical attention. In cases of severe diarrhea, rehydration is the mainstay and can be lifesaving when immediately initiated^2^.

Furthermore, for long-term effective control of cholera transmission, economic status and access to safe water are the mainstays. From here, the Wash, Sanitation, and Hygiene (WASH) action plan, a long-term implemented plan toward environmental conditions, was implemented to provide universal access to safe water and good hygienic conditions^24^,^26^.

Concerning cholera vaccination, it is available and recommended by the Centers for Disease Control and Prevention for travelers to, or for residents in countries where cholera is endemic. It is judicious to counsel a physician before the trip for a good travel plan. Providence of vaccine during humanitarian crisis predisposing to cholera and in times of outbreaks, is also advisable. Appropriate application of these simple measures, can reduce the risk of cholera^2^.

The re-emergence of cholera after a lengthy response against coronavirus disease 2019, in a country with an ongoing economic meltdown could indicate a crippling healthcare system^27^. Aside from the individual measures that can be taken to control the disease’s spread, a large responsibility falls on the government, especially during these times.

A larger percentage of the country’s GDP ought to be allocated toward healthcare. Important steps to be taken include assigning a larger number of hospital beds in public hospitals towards cholera patients. The investigation of the source of infection, particularly in the northern regions of Lebanon where cholera has been most prevalent. Furthermore, a negative stool examination should be required of all travelers entering Lebanon by land. The constant surveillance of water resources in high-risk areas should be maintained as well. This would allow the rapid containment of any recurrence.

The availability of medications and pharmaceutical products is an essential part of the response to any outbreak. Lebanon has one of the highest expenditures on pharmaceuticals in the Middle East^28^. These products are mostly imported from other countries (95%) at high costs. The depreciating currency has further worsened this issue by driving the medication prices even higher. The country, however, has 12 companies that are certified by the WHO yet are only functioning at 50% capacity. With the extra funding, the government could subsidize this local pharmaceutical industry to increase the production of high-quality and low-cost products^28^. Currently, subsidized medications are being smuggled out of the country via the black market^28^.

Consequently, the allocation and distribution of these products should be strictly overseen to prevent the former from happening.

## Conclusion

After 30 years since the last cholera epidemic in Lebanon back in 1993, the first confirmed cholera case in Lebanon was reported on 10 October 2022. Up until 29 December 2022, 699 confirmed cases and 23 confirmed deaths had been reported. The Ministry of Public Health is providing cooperation and support in containing the disease and covering the hospital and treatment expenses for cholera patients. Yet, more measures ought to be taken to strengthen the Lebanese healthcare system against the continuous threat communicable disease outbreaks.

## Ethics approval

Not applicable.

## Consent for publication

Not applicable.

## Sources of funding

We have not received any financial support for this manuscript.

## Authors’ contribution

O.U. and C.B.: conceptualization, project administration, writing – review and designing. J.W.: reviewed and edited the second draft. C.B.: reviewed and edited the first draft. O.U.: reviewed and edited the final draft. Data collection and assembly, manuscript writing, and final approval of manuscript done by all authors.

## Conflicts of interest disclosure

The authors declare no conflicts of interest.

## Research registration unique identifying number (UIN)

1. Name of the registry: not applicable.
2. Unique identifying number or registration ID: not applicable.
3. Hyperlink to your specific registration (must be publicly accessible and will be checked): not applicable.

## Guarantor

Olivier Uwishema, Principal Investigator (PI).

## Data availability statement

Not applicable.

## Provenance and peer review

Not commissioned, externally peer reviewed.
